# Incidence, Prevalence, and Survival Outcomes of Patients with Myeloproliferative Neoplasms in the United States: a SEER database analysis, years 2000–2021

**DOI:** 10.21203/rs.3.rs-9510524/v1

**Published:** 2026-05-06

**Authors:** Sachi Singhal, Rahul Mishra, Sankalp Arora, Noha Sharafeldin, Ruchi Desai, Tania Jain, Pankit Vachhani

**Affiliations:** 1 Department of Hematology/Oncology, Fox Chase Cancer Center, Philadelphia, PA; 2 Department of Internal Medicine, Luminis Health Anne Arundel Medical Center, Annapolis, MD; 3 Division of Cancer Medicine, UT MD Anderson Cancer Center, Houston, TX; 4 Division of Hematology and Oncology, Department of Medicine, University of Alabama at Birmingham, Birmingham, AL; 5 Department of Medicine, Division of Hematology, Oncology, and Palliative Care, Virginia Commonwealth University, Richmond, VA; 6 Division of Hematological Malignancies and Bone Marrow Transplantation, Sidney Kimmel Comprehensive Cancer Center, Johns Hopkins University, Baltimore, Maryland

**Keywords:** epidemiology, myelofibrosis, polycythemia vera, essential thrombocythemia, CML, PMF, ET, PV

## Abstract

Myeloproliferative Neoplasms (MPNs) are rare hematologic neoplasms. The epidemiology and outcomes of these rare neoplasms warrant an update considering new diagnostic tools and advances in treatment options. NCI’s Surveillance, Epidemiology and End Result (SEER)-17 registries were interrogated to analyze adult patients diagnosed with MPN [ICD-O-3 codes: polycythemia vera (PV) 9950/3, essential thrombocythemia (ET) 9962/3, primary myelofibrosis (PMF) 9961/3, and chronic myeloid leukemia (CML) 9875/3] during the years 2000 to 2021. Data of 63,242 (ET, n=24 172; PV, n= 23 456; PMF, n=6 131; CML, n=9 483) patients were analyzed. The overall incidence rate (IR) for ET, PV, PMF and CML was 1.8, 1.7, 0.5 and 0.7 per 100 000 person-years respectively. The incidence of ET, PV, and MF increased with age, and majority of cases were seen after the age of 50. 5-year Relative Survival (RS) for PMF was 50.9% in 2009, to 57.4% in 2013 and 60.6% in 2016. The median overall survival (OS) was 152 months for ET, 150 months for PV, 48 months for PMF, and 202 months for CML. Advancing age was associated with adverse survival across MPN subtypes. Survival of patients with PMF continues to improve over recent years. Important racial and gender variations exist were noted in our study.

## Introduction

Myeloproliferative neoplasms (MPNs) are classified into *BCR::ABL1* positive chronic myeloid leukemia (CML) and *BCR::ABL1* negative MPNs, which include polycythemia vera (PV), essential thrombocythemia (ET), primary myelofibrosis (PMF).^1^ These are collectively referred to as “classical” MPNs.^2^

The last couple of decades have witnessed remarkable diagnostic and therapeutic changes for all classical MPNs. The discovery and routine use of *BCR::ABL* testing for CML was an early oncologic triumph. Similarly, the discovery of JAK2 V617F (2005), MPL (2006), and CALR (2013) driver mutations of MPNs were landmark events.^3, 4^ Cumulatively, these advances were adopted in diagnostic criteria and allowed for diagnosis of occult or suspicious cases, potentially resulting in improved detection and higher diagnosis rates.^5–7^ From a management standpoint, the approval of imatinib in CML is a watershed moment in oncology.^8^ Following imatinib, multiple subsequent-generation TKIs (dasatinib, nilotinib, bosutinib, ponatinib, asciminib) were approved, further refining CML management.^9–13^ Similarly, the 2011 US FDA approval of ruxolitinib in myelofibrosis ushered in a new era.^14, 15^ It was also approved in 2015 for second line management of PV. Three other TKIs (fedratinib, pacritinib, and momelotinib) have also received approval in myelofibrosis while interferon has seen resurgence in MPNs – including the 2021 approval of ropeginterferon alpha-2b-njft for PV.^16–18^

With such defining events having occurred, we surmised that the epidemiology and outcomes of MPNs could be affected. Prior studies using data from the Surveillance, Epidemiology, and End Results (SEER) database from 2002–2016 have demonstrated an overall incidence of 1.57, 1.55, 0.44 per 100 000 person-years for PV, ET, and PMF respectively, with a rising incidence of ET over time. A decrease in mortality for PV and PMF was noted.^19^ On the other hand, the incidence of CML has been stable over the years, at about 1.3 per 100 000 person-years in 2016, with a striking decrease in mortality attributed to the advent of TKIs: 5-year relative survival (RS) of 83% in 2011, compared to 47% in 2001.^20, 21^ However, the epidemiology and survival outcomes data since 2016 is limited.^22, 23^ Therefore, we sought to update the incidence, prevalence, and outcomes of classical MPNs. We present the updated findings from the latest available SEER data on classical MPNs, with particular focus on the overall trends of incidence, prevalence, and survival outcomes over a two-decade period 2000–2021.

## Methods

### SEER database:

SEER database was used to estimate the incidence, prevalence, and survival outcomes of the classical MPNs during years 2000–2021 (www.seer.cancer.gov).^24^ Specifically, SEER 17 registries which capture data for cases diagnosed from 2000 through the current data year and include expanded races were utilized. This database represents 26.5% of the US population. For prevalence estimation only, SEER 8 registries were additionally queried since these have data starting from 1975 (compared to SEER 17 which starts in 2000).

### Patient data:

Deidentified data of patients aged ≥ 20 diagnosed with ET, PV, PMF, and CML between January 2000 to December 2021 were obtained from SEER database using the International Classification of Diseases for Oncology codes (ICD-O-3) codes: PV: 9950, ET: 9962, PMF: 9961, and CML: 9875. SEER analysis (e.g. incidence rates) is grouped in age categories of 5-years (e.g. 0–4, 5–9, and further). As these diagnoses are rare below 20, we chose age ≥ 20 to remain consistent with all analyses. Data obtained included patient demographics (age, sex, race/ethnicity), socioeconomic status (median household income, geographic distribution), year of diagnosis, survival status (alive/deceased), and time to death (if available). For comparison of data to the other more commonly treated malignancies, we retrieved similar data for patients with MDS and AML (ICD-O-3 codes listed in supplementary table 1).

### Statistical analysis:

Descriptive statistics were used to summarize patient characteristics. Incidence, survival, and prevalence were estimated using SEER*Stat software (version 8.4.4).

IR were stratified by gender and calendar year for age at diagnosis ≥ 20 years. Rates are age-adjusted to the 2000 US standard population. We assessed IRs overall and according to gender, year of diagnosis, and age at diagnosis. Sex-based IRRs were calculated as ratios of men-to-women IRs.

We calculated age and sex stratified prevalence percentage (percentage of total US population affected by MPN) of each disease subtype for patients alive on Jan 1, 2021, using the average of 2020 and 2021 census as the denominator total population. We estimated the total prevalence of these MPNs for entire US population by multiplying each MPN’s prevalence on Jan 1, 2021 with a conversion factor (Supplementary Table 2). Population pyramids were constructed to depict findings.

Relative Survival (RS) was defined as the ratio of the proportion of observed survivors in a cohort of cancer patients to the proportion of expected survivors in a comparable set of cancer-free individuals. RS adjusts for general survival of the US population (age, race, sex, and date at which age was coded). 1-, 5- and 10-year RS with associated confidence intervals were reported. Kaplan-Meier survival analysis was conducted to assess OS and log rank test was used to compare subgroups. Median survival was estimated using the reverse Kaplan-Meier method.

## Results

### Baseline characteristics:

**Figure 1:**
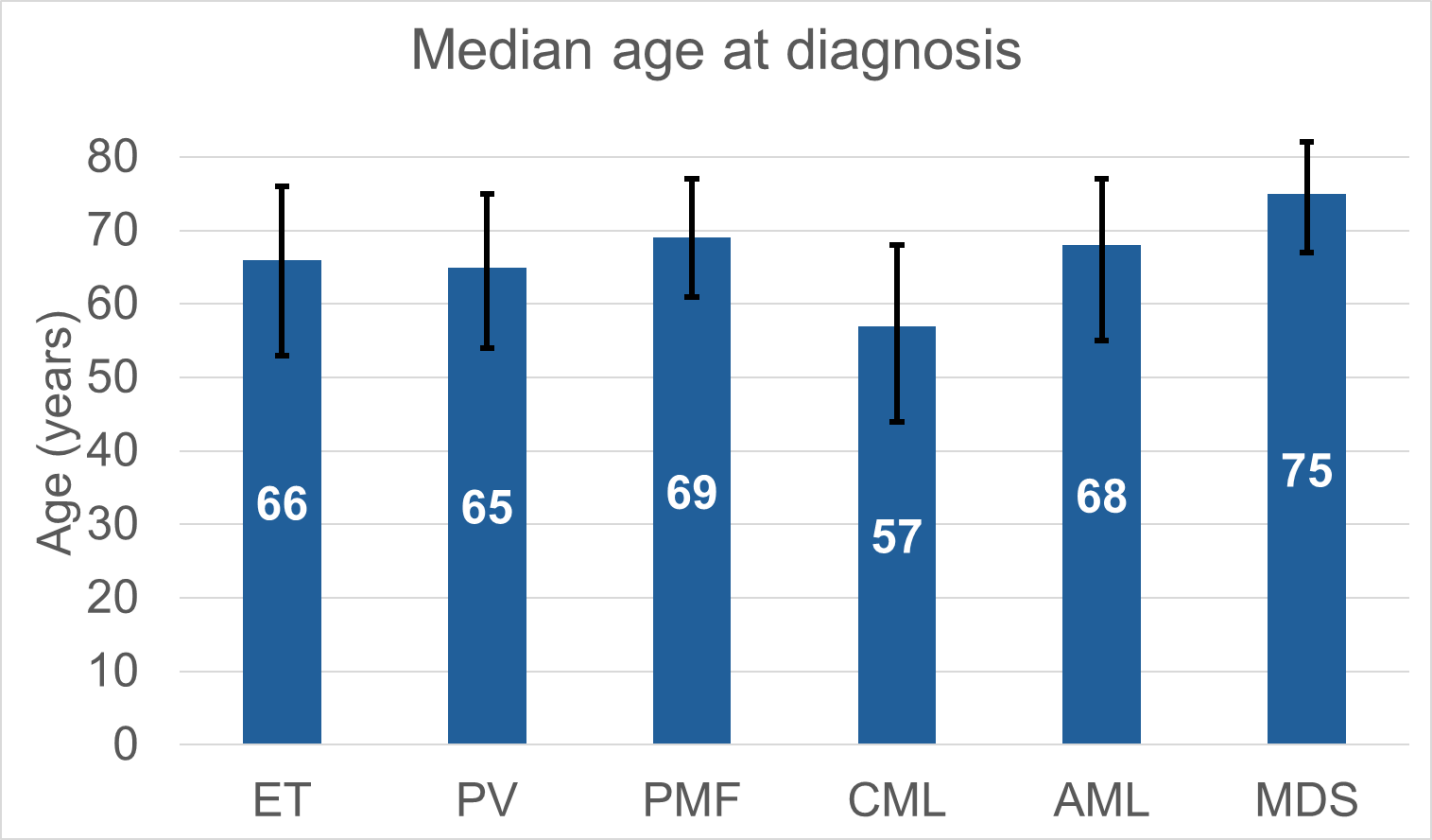
Median age of diagnosis with myeloid neoplasms in the United States between years 2001–2021. AML: acute myeloid leukemia, CML: chronic myeloid leukemia, ET: essential thrombocythemia, MDS: myelodysplastic syndromes, PMF: primary myelofibrosis, PV: polycythemia vera

A total of 63 242 (ET, n=24 172; PV, n=23 456; PMF, n=6 131; CML, n=9 483) patients were evaluated (Table 1). The population was evenly distributed in terms of sex, with women comprising 50% (n=31 649) of this cohort. The median age of patients for ET, PV, PMF, and CML groups was 66 (IQR 53–76), 65 (IQR 54–75), 69 (IQR 61–77) and 56 (IQR 43–68) years respectively (Figure 1). Non-Hispanic Whites (NHW) comprised 70% ET, 77% PV, 75% PMF and 65% of patients with CML respectively (Figure 1). Racial breakdown for individual MPNs is shown in Supplementary Figure 1.

Socio-economic and geographic distributions are detailed in Supplementary Table 3. Median household income was available for almost the entire cohort (~99% patients). Notably, 6.7% of the cohort lived in households earning <$50 000 annually. A majority (62.5%) of patients lived in metropolitan areas with population >1 million.

### Incidence Rates (IR):

The overall IR for ET, PV, PMF, and CML were 1.84, 1.78, 0.47 and 0.72 per 100 000 person-years respectively (supplementary Table 4). The age adjusted IR in patients of age 60 or more was the highest for ET (4.67 per 100 000 person-years), followed by PV (4.56 per 100 000 person-years), PMF (1.44 per 100 000 person-years) and the lowest for CML (1.30 per 100 000 person-years). In patients <60 years, ET and PV had comparable IR (0.76 an 0.74 per 100 000 person-years respectively) followed by CML and PMF (0.5 and 0.12 per 100 000 person-years respectively). Sex-based IR variations were prominent in patients ≥60; ET predominated in women (IR 5.20), while PV predominated in men (IR 5.40) (Supplementary Table 4).

**Figure 2:**
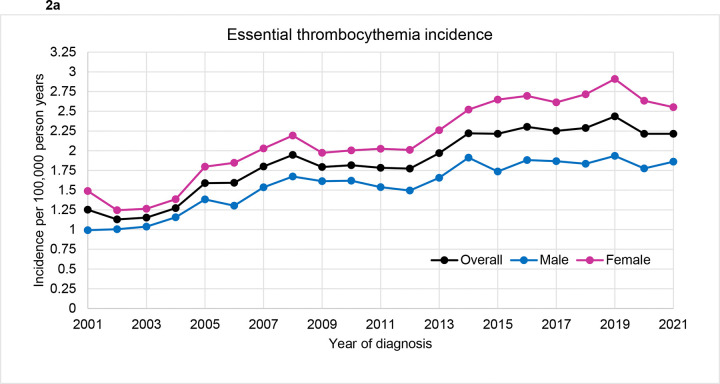

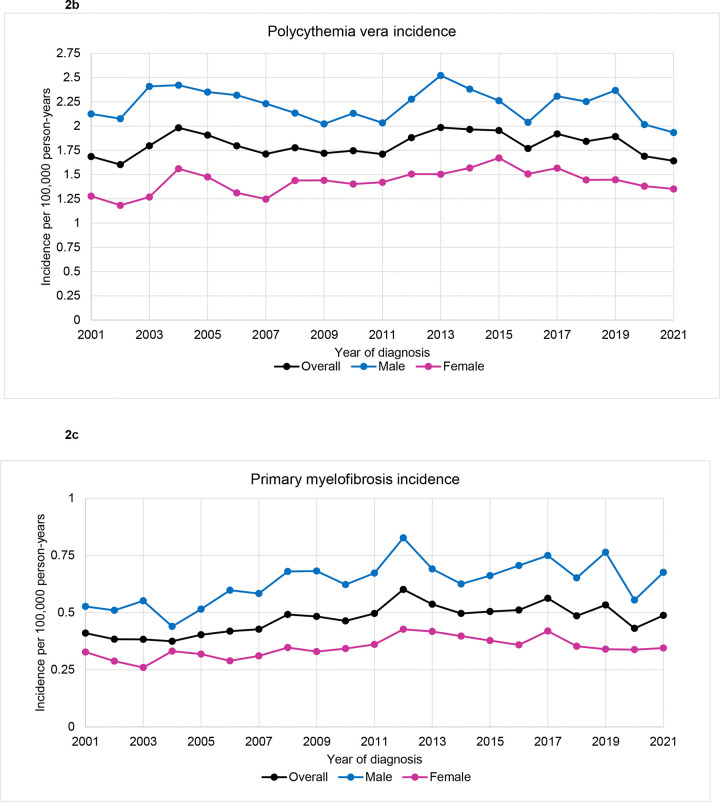

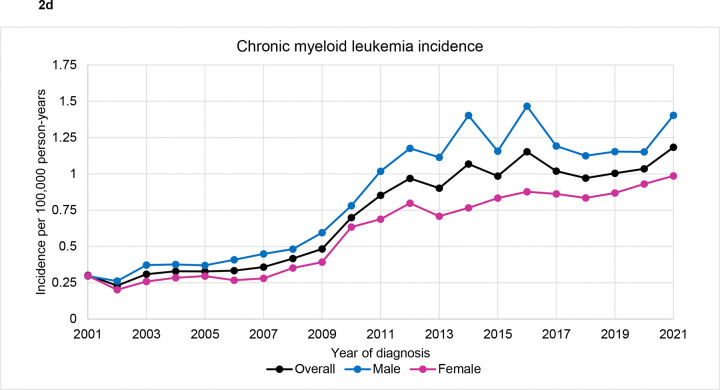
Incidence rate (IR) by year of diagnosis and gender for those diagnosed between years 2001 and 2021 for (a) ET: essential thrombocythemia, (b) PV: polycythemia vera (c) PMF: primary myelofibrosis, and (d) CML: chronic myeloid leukemia. The overall and 2021 specific IR were as follows: ET (1.84, 2.21 per 100,000 person-years), PV (1.78, 1.64 per 100,000 person-years), PMF (0.47, 0.48 per 100,000 person-years), and CML (0.72, 1.18 per 100,000 person-years)

In 2001, the overall incidence rate of ET was 1.25 per 100 000 person-years, with more women being affected by ET than men (1.48 versus 0.99 per 100 000 person-years). (Figure 2a). The incidence rate increased over the years and was noted to be 1.95 per 100 000 person-years in 2008, after which it remained stable till 2013. It has been gradually increasing since then, with the highest incidence rate reported in 2019 (2.43 per 100 000 person-years). The most recent incidence rates from 2021 were 2.21 per 100 000 person-years overall, 2.55 per 100 000 person-years for women, and 1.86 per 100 000 person-years for men. Throughout 2001–2021, more women than men cases of ET were reported. The incidence rates of PV have been stable over the years, with IR of 1.69 per 100 000 person-years (2.12 and 1.28 per 100 000 person-years for men and women respectively) in 2001 and 1.64 per 100 000 person-years (1.93 and1.35 per 100 000 person-years for men and women respectively) in 2021 (Figure 2b). The incidence of PMF is generally low, with IR of 0.41 per 100 000 person-years noted in the year 2001 and has remained stable ever since. (Figure 2c) The most recent reported incidence rate in 2021 was 0.49 per 100 000 person-years overall, 0.68 and 0.35 per 100 000 person-years for men and women respectively. The incidence rates of CML cases were stable between the years 2001 and 2007, after which a consistent rise in reported cases was seen till the year 2012. (Figure 2d) Most recent rates include IR of 1.18 per 100 000 person-years overall, 1.40 and 0.99 per 100 000 person-years for men and women respectively.

**Figure 3:**
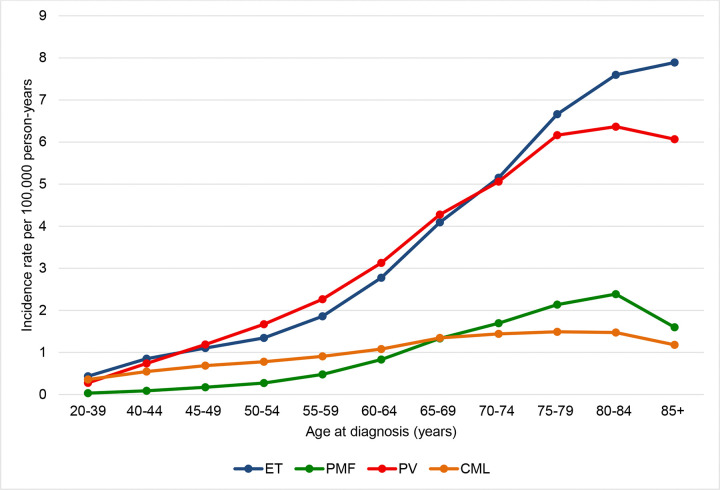
Incidence rate (IR) by age at diagnosis for patients diagnosed between years 2001 and 2021 with (a) ET: essential thrombocythemia, (b) PV: polycythemia vera (c) PMF: primary myelofibrosis, and (d) CML: chronic myeloid leukemia

The incidence of all classical MPNs increased with age in this population (Figure 3). Most cases were seen after the age of 50. Of note, the IR rates for patients aged 20–39 (otherwise termed as young adults) were 0.42, 0.26, 0.03 and 0.34 per 100 000 person-years for ET, PV, PMF and CML respectively. The IR for ET and PV was highest in the population over age 85 (7.9 and 6.1 per 100 000 person-years respectively). The incidence rates of CML were highest in ages 75–85 (1.5 per 100 000 person-years), and for PMF at age 80–85 (2.4 per 100 000 person-years). Gender variations existed in incidence rates across age groups. Incidence Rate Ratio (IRR) defined as IR for women divided by IR for men was noted to increase with age for PV and decrease with age for ET patients respectively. Further details can be found in supplementary figure 2.

### Prevalence:

Given the longer follow-up duration of patients in SEER 8 database, we utilized SEER 8 registry to report prevalence of patients living with MPNs. As on Jan 1, 2021, the overall prevalence of ET, PV, PMF and CML was 25.0, 21.2, 3.4, and 11.5 per 100 000 persons respectively (Supplementary Table 5). The population in SEER 8 database represents 8.3% of the total US population. Applying the conversion factor estimates (see Supplementary Table 2) on population census from July 2024, we estimated a total of 64 641 patients with ET, 54,604 patients with PV, 8 678 patients with PMF, and 29 716 patients with CML aged ≥ 20 years in the US.

**Figure 4:**
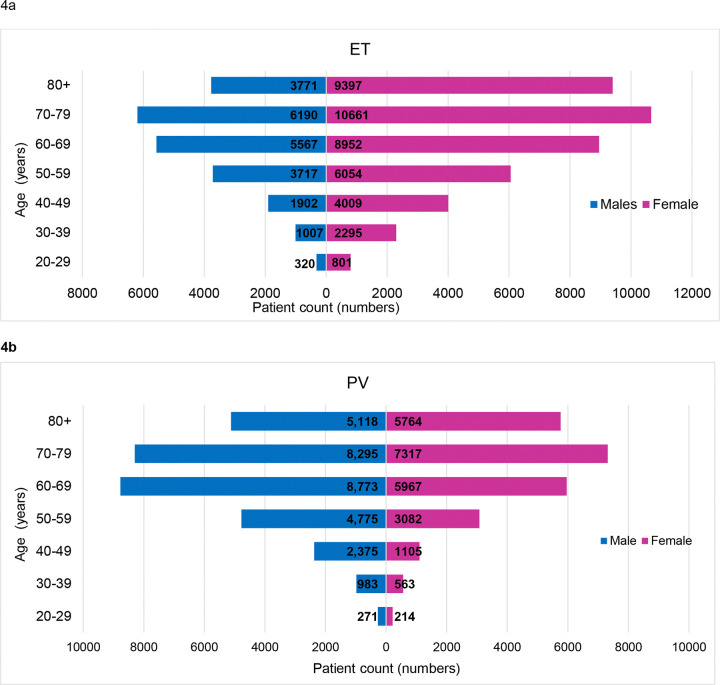

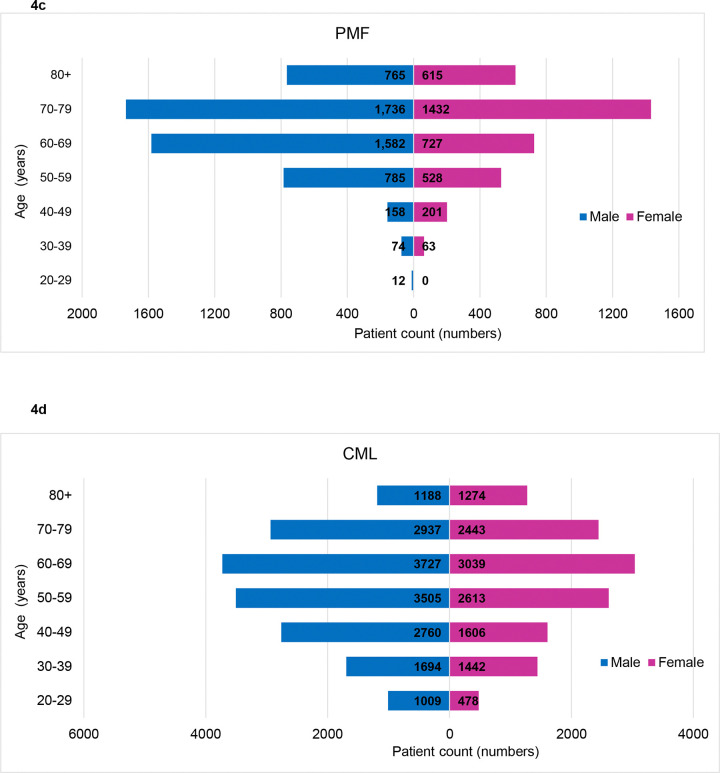
Population pyramids depicting estimated prevalence on Jan 1, 2024 using SEER 8 registries. (a) ET: essential thrombocythemia, (b) PV: polycythemia vera (c) PMF: primary myelofibrosis, and (d) CML: chronic myeloid leukemia

ET was more prevalent in women than men across all age groups. (Figures 4) Women in the age group 70–79 years were seen to have the highest prevalence (n=10 661). This is contrast to the age and gender distribution of patients living with PV, where the highest prevalence was noted in men aged 60–69 years (n=8773). PV demonstrated a male preponderance across all age groups except those above 80 years. PMF and CML were also estimated to be more prevalent in men, with the highest prevalence estimated in men aged 70–79 (n=1736) and 60–69 (n=3727) respectively. The population distribution for the estimated overall prevalence by age and gender for United States, estimated for population estimates from July, 2024 can be noted in Figure 4. Population distribution utilizing the SEER 17 database can be found in supplementary Figure S5.

### Relative survival:

**Figure 5:**
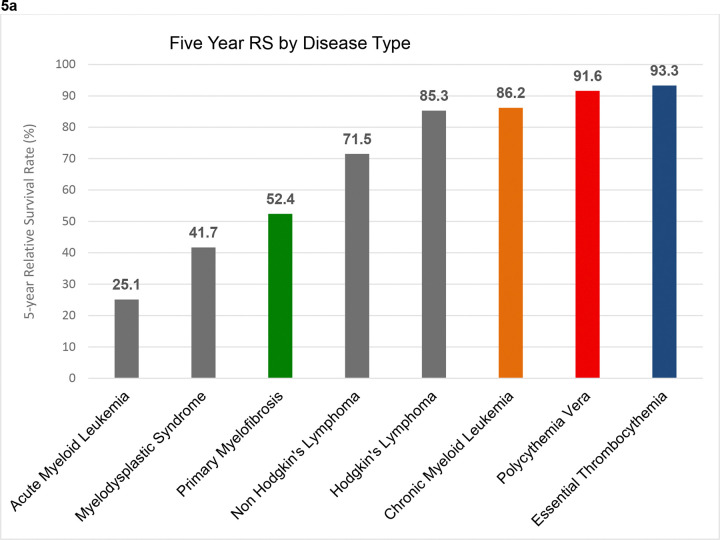

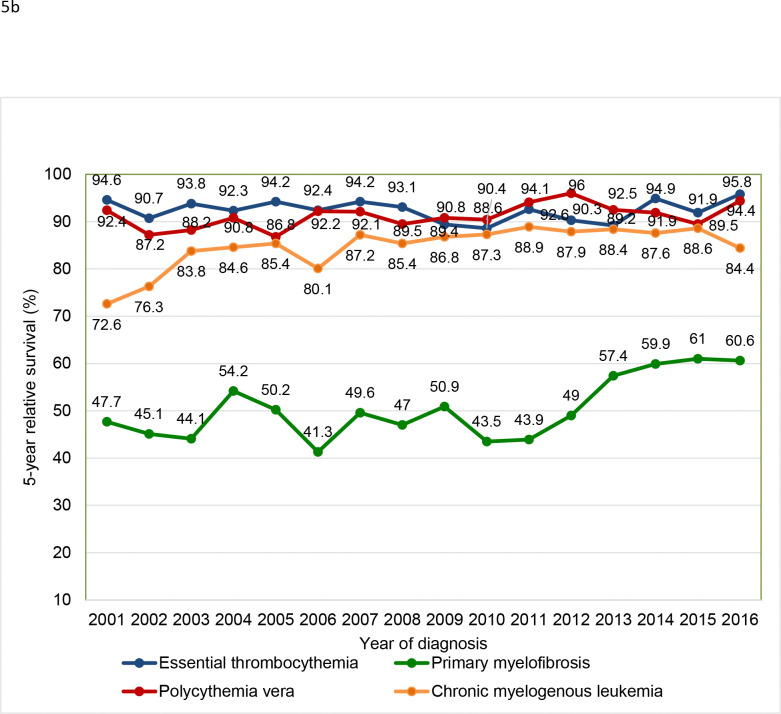

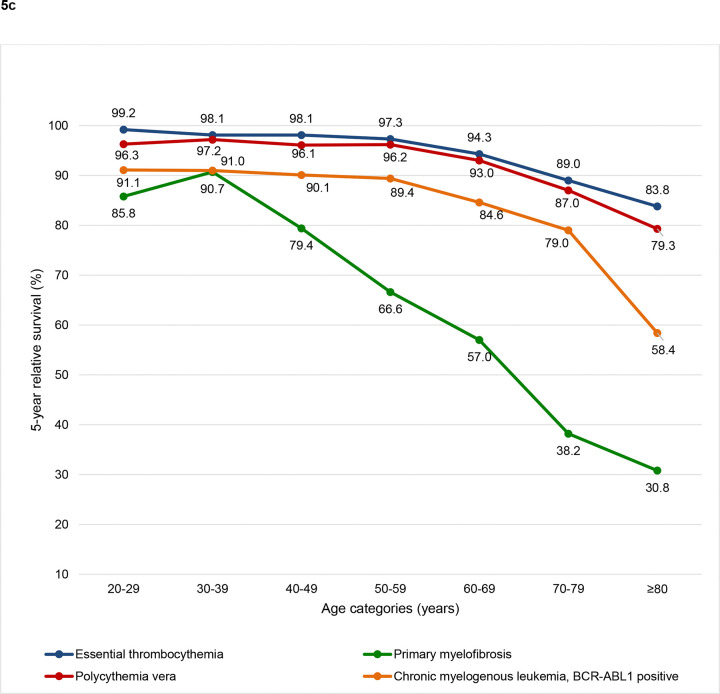

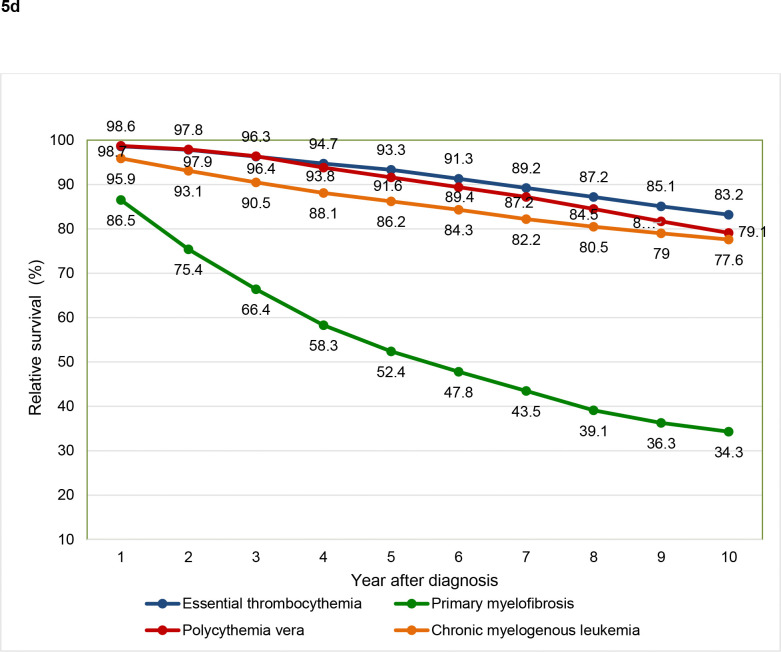
Relative Survival (RS) for classical MPNs in the 2001–2016 period, followed to the end of 2021. (a) 5-yr RS for MPNs and other select hematologic malignancies in the United States, (b) 5-yr RS for MPNs assessed by year of diagnosis, (c) 5-yr RS for MPNs assessed by age at diagnosis, (d) RS trend from 1 to 10 yrs since diagnosis of MPNs.

5-year RS for PV, ET, PMF, and CML were 91.60%, 93.30%, 52.40%, and 86.20% respectively (Figure 5a). This contrasts with AML and MDS, where patients had a 5-year RS of 25% and 41% respectively. The 10-year RS of PV, ET, and CML were 79.1%, 83.2%, and 77.6% respectively (Figure 5b). The RS for all classical MPNs declined over time (Figure 5d). This was most notable for PMF where it declined from 86.5% at year 1 to 34.3% at year 10.

When analyzed by year of diagnosis (Figure 5c), ET and PV maintained consistently favorable 5-year RS throughout the study period (Figure 5c). RS for patients living with PMF showed a continuous improvement from years 2009 and onwards. 5-year RS for PMF was 50.9% in 2009 to 57.4% in 2013 and 60.6% in 2016. For CML, the 5-year RS showed a marked improvement between years 2001 (72.6%) and 2005 (85.4%); since then, it has been consistently stable around 88% with minor fluctuations.

The 5-year RS of all classical MPNs showed a decline with advancing age at diagnosis. (Figure 5c). Patients living with PMF consistently did worse than other MPNs across ages ≥ 40. In patients with age ≥ 80 years, the 5-year RS of patients living with ET was 83.8%, PV 79.3%, CML 58.4%, and PMF 30.8%.

### Overall Survival (OS)

The median overall survival was 152 months (95% CI: 149–155 mos) for ET, 150 months (95% CI: 147–153 mos) for PV, 48 months (95% CI: 46–50 mos) for PMF, and 202 mos (95% CI: 187–216 mos) for CML (Figure 7).

**Figure 6:**
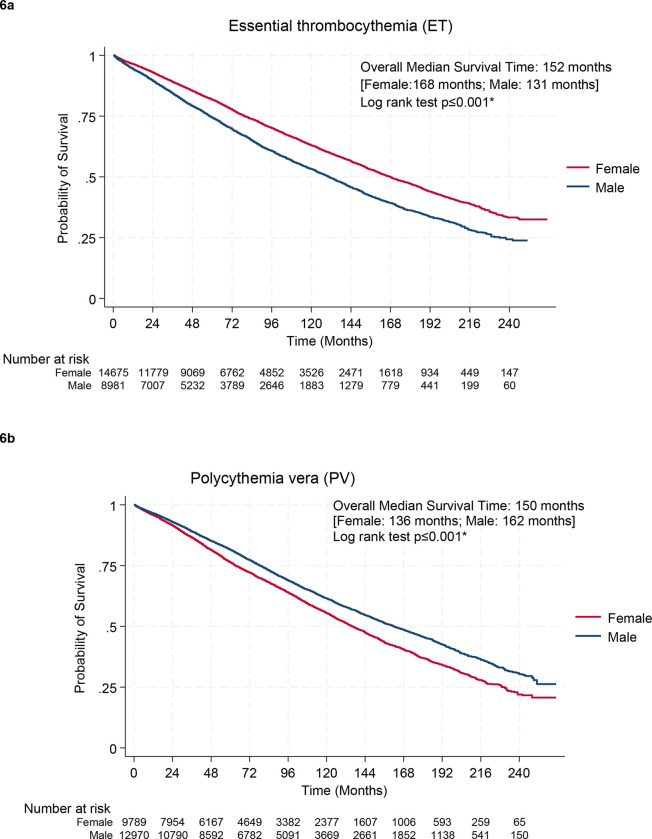

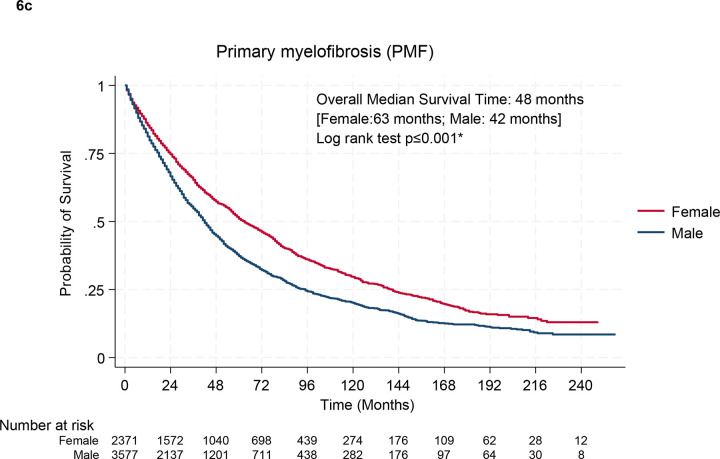

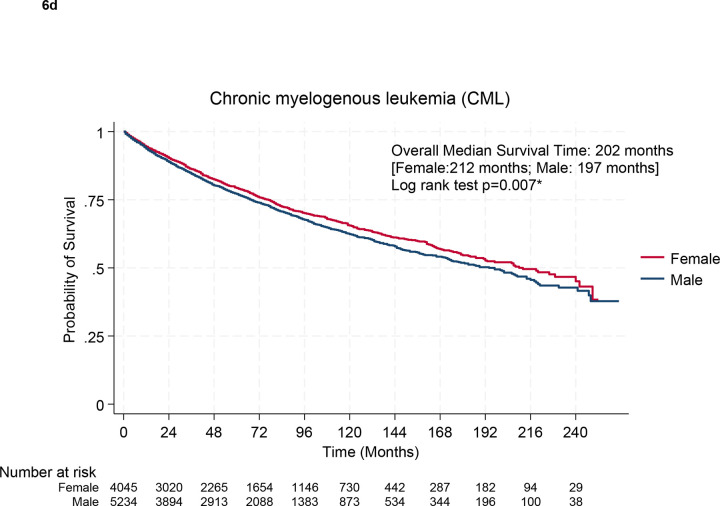
Overall survival for MPNs by gender for (a) ET: essential thrombocythemia (b) PV: polycythemia vera (c) PMF: primary myelofibrosis, and (d) CML: chronic myeloid leukemia.

**Figure 7:**
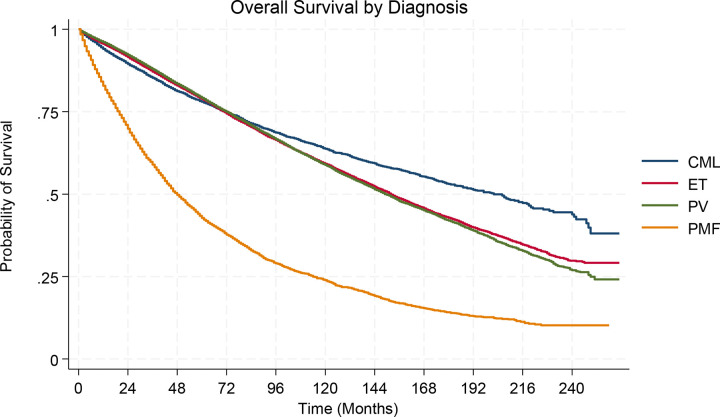
– Overall Survival curves for patients living with Myeloproliferative Neoplasms in the United States between 2000–2021(a) ET: essential thrombocythemia (b) PV: polycythemia vera (c) PMF: primary myelofibrosis, and (d) CML: chronic myeloid leukemia.

When stratified by age at diagnosis, advancing age was associated with adverse survival across MPN subtypes (Supplementary Figure 6). Women with ET, (Figure 6a; median OS 168 mos, 95% CI: 163–172 mos, vs median OS 131 mos, 95% CI: 126–135 mos, p <0.001). PMF (Figure 6c median OS 63 mos, 95% CI: 59–68 mos versus 42 mos, 95% CI: 40–44 mos, p <0.001) and CML (Figure 6d median OS 212 mos, 95% CI 192–242 mos, versus median OS 197 months, 95% CI 175–214 months, p =0.0073) had a significantly better OS than men. In contrast, a significantly higher OS was seen in men living with PV as compared to women (Figure 6b) median OS 162 mos, 95% CI: 157–167 mos vs. 136 mos, 95% CI: 132–140 mos, p <0.001).

OS varied by the year of diagnosis as well. (Supplementary Figures 10). Patients diagnosed in 2011 or later did better. An improved OS was observed in patients diagnosed between the years 2018–2021, as compared to years 2011–2017 as well. (Supplementary Figure 10c). Survival differences were also seen between racial sub-groups in each MPN cohort. Further details can be found in Supplementary Figure 6 and 7.

## Discussion

Our study found that the incidence, prevalence, and survival of patients living with MPNs continues to increase over time, with some differences seen based on age, gender, racial, and socio-demographic factors.

The overall IRs for ET, PV, and PMF in our study were 1.8, 1.7 and 0.5 per 100 000 person-years respectively. When compared to prior studies that captured data until 2016, the IRs of all Philadelphia chromosome negative MPNs have increased.^19, 21^ The highest increase in overall IR was noted in ET cases (from 1.55^4^ in 2016 to 1.8 per 100 000 person years^19^), with the most recent 2021 IR for ET being 2.2 person years. Like prior SEER analyses, the incidence of ET was noted to be higher in women compared to men. This contrasts with European studies, which reported higher incidence of ET in men than women.^25, 26^ On the other hand, the incidence of both PV and MF was higher in men. The IR rate of CML in our cohort was 0.7 person years, which is lower than the 1.3 person years reported in prior SEER analyses. This is likely explained by methodology. We used only ICD code 9875 (chronic myelogenous leukemia, BCR/ABL positive) for CML, which became reportable starting year 2000. We report in our study a fourfold increase in prevalence of this particular type of CML. This contrasts with prior studies where ICD codes 9875 and 9863 were combined for analysis^21^. ICD-0–3 9863 represents CML, NOS which may have been used broadly to capture various myeloid malignancies including MDS/MPN overlap syndrome entities. Sasaki et al.^27^ had previously shown a marked difference in OS between Philadelphia chromosome positive CML and CML-NOS, indicating the latter was a heterogeneous grouping and therefore one to avoid in epidemiologic studies of CML.

We also noted an increase in prevalence of all the classical MPNs compared to prior SEER based study estimates.^28^ To our knowledge, we are the first to report an age-group and gender based estimated prevalence for each MPN from a large database. The population pyramids show a clear difference in the distribution of patients between the MPNs. Overall, the estimated total number of ET and PV patients age ≥ 20 years of age utilizing prevalence estimates from SEER8 are 64 641 and 54 604 respectively. These are broadly consistent with regional US database estimates.^29, 30^ On the other hand, even though the prior study’s MF prevalence (PMF, post-ET MF, and post-PV MF; 2.12–3.8 persons per 100 000) seems comparable to our PMF prevalence (3 persons per 100 000), of note, SEER does not allow for a reliable capture of post-ET and post-PV MF cases. The prevalence for CML increased fourfold from 2006 to 2021 – likely explained by increased incidence, correct diagnosis and ICD code selection, and decreasing mortality.

There has been a significant improvement in OS of CML over the years. A median OS of 16.8 years was noted for CML patients in our cohort, which is significantly higher than previous reports, with women doing slightly better than male counterparts.^31, 32^ No change in OS trends was noted with the advent of second and third generation TKIs in CML patients as a whole – perhaps unsurprising as OS advantage of second or third generation TKIs over imatinib has not been shown. Survival outcomes for PMF remain adverse with a median OS of 48 months. There appears to be an improvement though in the more recent cohorts as compared to the older cohort of PMF patients. Similar improvement in PMF survival was reported previously by others as well^19^. These findings contrast with studies analyzing outcomes prior to the year 2010^21, 33^. Many factors may be contributing to improved survival in recent years, perhaps most importantly treatment with JAK inhibitors – primarily ruxolitinib for our study^19, 34, 35^.

Our analysis demonstrated that women with ET had significantly longer overall survival compared to men. Large population based study from the Mayo Clinic and the University of Florence, Italy have previously demonstrated male sex being an independent negative prognostic risk factor with a HR of 1.6.^36^ Prior studies have noted other clinical differences as well, with women reporting a higher overall symptom score and men being more likely to have a history of blood transfusions.^37^ In fact, male gender is one of the risk factors for OS in the MIPSS-ET and Triple A Plus (AAA+) models^38, 39^. It’s unclear if it was specifically looked in to for the IPSET model^40^; for AAA model, male sex was a risk-factor in only one out of two cohorts and therefore not incorporated in the overall model^41^. Notably, sex was not a risk factor for OS in the MIPSS-PV model^38^. Better survival in women with PMF has also been reported in literature. A retrospective study from Mayo Clinic examined long term survivors of PMF and reported female gender as an independent variable (p=0.03) associated with survival beyond 20 years.^42^ However, sex does not make it to any of the traditional risk stratification models in MF. Slight gender variations occur in overall survival of CML patients as well, with women doing slightly better than men in our cohort, consistent with prior reports.^21^ Data has emerged in recent years signaling gender may play a role in gene expression and clonal expansion in MPNs. Biological factors, including sex-specific differences in JAK2 V617F allele burdens and gene expression, may underpin observed survival variations.^43,44^ Consistent with prior research, our data also suggests that gender may have a role in influencing overall outcomes in patients living with MPNs, and further studies are warranted to establish a correlation.^45, 46^

We also looked at relative survival to isolate the mortality risk from each MPN by accounting for other causes of death. For PMF, we found 5-year RS of 52.4% and a 10-year RS of 34.3%. The rapid drop in RS in MF patients over time from diagnosis shows the corroding effect of diagnosis on survival. This is in sharp contrast to PV, ET, and CML where the 5-yr RS was ~90% and 10-year RS was ~85%. The less than 100% RS for these three conditions – frequently viewed as “indolent” malignancies - nevertheless underscores the point that these too impact survival. The negative impact of age of diagnosis on RS was seen for all MPNs with a particularly sharp and continuous drop for MF beyond age 40 and for PV, ET, and CML beyond age 60. For CML diagnosed beyond age 80, the RS was < 60% - probably a reflection of lack of treatment, early discontinuations, or complications. That said, a notable positive uptrend in RS was noted for MF patients in recent years, rising from 43.5% in 2010 to 61% in 2015 likely a result of ruxolitinib treatment.

Significant racial disparities were noted in our cohort. NHW population was noted to have an inferior survival compared to other racial groups in both PV and ET. This may in part be explained by a previous study that reported Caucasian patients have a higher risk of progression to myelofibrosis.^47^ Our findings are in contrast with prior studies which report shorter median survival time for non-White patients living with MPNs.^48^ An important point to note here is that we examined racial sub-groups in greater detail, with distribution into NHB, NHW, Hispanic, AI/AN, AAPI and other subgroups, instead of “White vs Non-White” which may explain some unique findings in our cohort compared to previous studies. Previous studies have often excluded and/or clubbed racial minorities.^49^ Our finding is consistent with another recent SEER-based study, which also reported A/APIs living with PMF did significantly better.^50^ Interestingly, they noted that this finding was present even after adjustment for other socio-demographic determinants such as age, sex, income; marital status and year of diagnosis, raising the possibility that the differences may lie, at least in part, in biological factors. It is important to note here that previous studies have noted no racial variation in the primary mutation landscape of PMF (70% JAK2, 18% CALR, 5% MPL).^51^

Socio-economic disparities may play an important role in incidence and outcomes of patients living with MPNs. The MOSAICC^52^ study also noted increasing low childhood socioeconomic status (OR 2.30, 95% CI 1.02–5.18) as an important risk factor of developing MPNs. Previous studies have noted a significant financial and psychosocial burden of disease associated with MPNs.^53–55^ Studies have demonstrated that lower median income may be an independent negative prognostic marker of overall survival^56^. In our analysis, a significant proportion (6.7%) of patients lived with a median household income < $50 000, and (6.3%) lived in a non-metropolitan area not adjacent to a metropolitan area. This may pose a barrier to expensive medications, transport to health care facilities, enrollment on clinical trials, or management plans – impacting this traditionally ‘underserved’ population’s outcomes negatively.

Our study has several strengths and limitations. Probably the biggest strength is the large volume of cases of each of the MPNs in the SEER database – making this one of the largest MPN epidemiological and outcomes study. It also afforded reliable calculations of IR, prevalence, and survival statistics for each MPN as well as its gender, age, or racial subgroups. That the data comes from SEER database which captures a diverse set of patients spanning various geographic and socio-economic statuses makes the study findings applicable to the overall US population. In addition, our study captures a wide timespan – allowing to assess trends in epidemiological shifts in the background of changing diagnostics and therapeutic landscape. That said, the inherent issues with SEER database also pose limitations: chief amongst these is the possibility of misclassification of diseases. This is a problem for Philadelphia chromosome negative MPNs due to the overlap in clinical presentation, laboratory, molecular, and even morphological features^57^. Resultant inter-observer variability, with approximately 80% agreement rate, in establishing a specific subtype of MPN diagnosis has been noted previously^21, 26, 58–60^. The importance of bone marrow histopathologic evaluations cannot be overstated for accurate MPN diagnosis. However, with increasing utilization of sensitive peripheral blood molecular testing of MPN driver mutations, bone marrow biopsy evaluations are increasingly omitted. This was previously reported by Srour et al. and our updated data (supplementary table S6, figure S10) confirms the trend. In addition, under ascertainment and to a lesser degree underreporting of cases especially in the elderly and/or minority group populations could also downsize the overall incidence and prevalence statistics. Similarly, delayed diagnosis in these populations may contribute to worse outcomes. Finally, the SEER program allows a standard delay of 22 months between the end of the diagnosis year and first reporting. Delayed reporting refers to addition of cases or new information received beyond the standard 22-month delay. Srour et al.^49^ had previously reported 18–36% and 15–49% higher IRs for PV and ET respectively with delayed reporting. However, given the nearly two-decade long period in our study, the effects of delayed reporting should be minimal.

## Supplementary Material

Supplementary Files

This is a list of supplementary files associated with this preprint. Click to download.

• SupplementaryfinalsubmissionSSPV1.pdf

• TablesandfiguresformainmanuscriptfinalsubmissionSSPV.docx

## Figures and Tables

**Table 1:** Baseline characteristics of patients with myeloproliferative neoplasms (MPNs) diagnosed between years 2001–2021.

Variable	ET	PV	PMF	CML	Overall

Total	24172 (38%)	23456 (37%)	6131 (9.7%)	9483 (15%)	63242 (100%)

Age (median at diagnosis) IQR	66 (53–76)	65 (54–75)	69 (61–77)	56 (43–68)	

Sex; n (%)					
F	4971 (62%)	10118 (43%)	2444 (40%)	4116 (43%)	31649 (50%)
M	9201 (38%)	13338 (57%)	3687 (60%)	5367 (57%)	31593 (50%)

Year of Diagnosis; n (%)					
2000–2005	3354 (14%)	4729 (20%)	1013 (17%)	828 (9%)	
2006–2010	5082 (21%)	5046 (22%)	1299 (21%)	1339 (14%)	
2011–2016	7790 (32%)	7302 (31%)	2011 (33%)	3754 (40%)	
2017–2021	7945 (33%)	6379 (27%)	1808 (29%)	3562 (37%)	

Alive	15942 (66%)	14393 (62%)	2209 (36%)	6903 (73%)	39447 (63%)
Deceased	8230 (34%)	9063 (38%)	3922 (64%)	2580 (27%)	23795 (37%)

CML: Chronic myeloid leukemia BCR:ABL positive, ET: essential thrombocythemia, F: women, IQR: inter-quartile range, M: men, PMF: primary myelofibrosis, PV: polycythemia vera,)

## Key Points

- The incidence of ET, PV, PMF, and CML are increasing, with an overall incidence rate of 1.8, 1.7, 0.5, and 0.7 per 100 000 person-years respectively
- The prevalence of ET, PV, PMF, and CML are 25.0, 21.2, 3.4, and 11.5 per 100 000 persons respectively
- The median overall survival ET, PV, PMF, and CML are 152 months, 150 months, 48 months, and 202 months
- All MPNs demonstrated decreased 5-year relative survival compared to the general population
- Age, gender, and race-based differences exist for incidence, prevalence, and survival outcomes of all MPNs

## References

[1] Khoury JD, Solary E, Abla O, The 5th edition of the World Health Organization Classification of Haematolymphoid Tumours: Myeloid and Histiocytic/Dendritic Neoplasms. Leukemia. 2022;36(7):1703–1719. 35732831 10.1038/s41375-022-01613-1 PMC9252913

[2] Thapa B, Fazal S, Parsi M, Rogers HJ. Myeloproliferative Neoplasms. StatPearls. Treasure Island (FL): StatPearls Publishing Copyright © 2025, StatPearls Publishing LLC., 2025. 30285359

[3] James C, Ugo V, Le CouØdic JP, A unique clonal JAK2 mutation leading to constitutive signalling causes polycythaemia vera. Nature. 2005;434(7037):1144–1148. 15793561 10.1038/nature03546

[4] Thiele J, Kvasnicka HM. The 2008 WHO diagnostic criteria for polycythemia vera, essential thrombocythemia, and primary myelofibrosis. Curr Hematol Malig Rep. 2009;4(1):33–40. 20425436 10.1007/s11899-009-0005-6

[5] Pikman Y, Lee BH, Mercher T, MPLW515L is a novel somatic activating mutation in myelofibrosis with myeloid metaplasia. PLoS Med. 2006;3(7):e270. 16834459 10.1371/journal.pmed.0030270 PMC1502153

[6] Klampfl T, Gisslinger H, Harutyunyan AS, Somatic Mutations of Calreticulin in Myeloproliferative Neoplasms. New England Journal of Medicine. 2013;369(25):2379–2390. 24325356 10.1056/NEJMoa1311347

[7] Nangalia J, Massie CE, Baxter EJ, Somatic *CALR* Mutations in Myeloproliferative Neoplasms with Nonmutated *JAK2*. New England Journal of Medicine. 2013;369(25):2391–2405. 24325359 10.1056/NEJMoa1312542 PMC3966280

[8] Druker BJ, Guilhot F, O’Brien SG, Five-Year Follow-up of Patients Receiving Imatinib for Chronic Myeloid Leukemia. New England Journal of Medicine. 2006;355(23):2408–2417. 17151364 10.1056/NEJMoa062867

[9] Kantarjian H, Shah NP, Hochhaus A, Dasatinib versus Imatinib in Newly Diagnosed Chronic-Phase Chronic Myeloid Leukemia. New England Journal of Medicine. 2010;362(24):2260–2270. 20525995 10.1056/NEJMoa1002315

[10] Saglio G, Kim D-W, Issaragrisil S, Nilotinib versus Imatinib for Newly Diagnosed Chronic Myeloid Leukemia. New England Journal of Medicine. 2010;362(24):2251–2259. 20525993 10.1056/NEJMoa0912614

[11] Cortes JE, Kim D-W, Kantarjian HM, Bosutinib Versus Imatinib in Newly Diagnosed Chronic-Phase Chronic Myeloid Leukemia: Results From the BELA Trial. Journal of Clinical Oncology. 2012;30(28):3486–3492. 22949154 10.1200/JCO.2011.38.7522 PMC4979199

[12] Hochhaus A, Wang J, Kim D-W, Asciminib in Newly Diagnosed Chronic Myeloid Leukemia. New England Journal of Medicine. 2024;391(10):885–898. 38820078 10.1056/NEJMoa2400858

[13] Cortes JE, Kantarjian H, Shah NP, Ponatinib in Refractory Philadelphia Chromosome–Positive Leukemias. New England Journal of Medicine. 2012;367(22):2075–2088. 23190221 10.1056/NEJMoa1205127 PMC3777383

[14] Verstovsek S, Mesa RA, Gotlib J, A Double-Blind, Placebo-Controlled Trial of Ruxolitinib for Myelofibrosis. New England Journal of Medicine. 2012;366(9):799–807. 22375971 10.1056/NEJMoa1110557 PMC4822164

[15] Harrison C, Kiladjian J-J, Al-Ali HK, JAK Inhibition with Ruxolitinib versus Best Available Therapy for Myelofibrosis. New England Journal of Medicine. 2012;366(9):787–798. 22375970 10.1056/NEJMoa1110556

[16] Harrison CN, Mesa R, Talpaz M, Efficacy and safety of fedratinib in patients with myelofibrosis previously treated with ruxolitinib (FREEDOM2): results from a multicentre, open-label, randomised, controlled, phase 3 trial. The Lancet Haematology. 2024;11(10):e729–e740. 39265613 10.1016/S2352-3026(24)00212-6

[17] Mascarenhas J, Gerds AT, Kiladjian J-J, PACIFICA: A Randomized, Controlled Phase 3 Study of Pacritinib Versus Physician’s Choice in Patients with Primary or Secondary Myelofibrosis and Severe Thrombocytopenia. Blood. 2022;140(Supplement 1):9592–9594.

[18] Verstovsek S, Gerds AT, Vannucchi AM, Momelotinib versus danazol in symptomatic patients with anaemia and myelofibrosis (MOMENTUM): results from an international, double-blind, randomised, controlled, phase 3 study. The Lancet. 2023;401(10373):269–280. 10.1016/S0140-6736(22)02036-0 36709073

[19] Verstovsek S, Yu J, Scherber RM, Changes in the incidence and overall survival of patients with myeloproliferative neoplasms between 2002 and 2016 in the United States. Leukemia & Lymphoma. 2022;63(3):694–702. 34689695 10.1080/10428194.2021.1992756

[20] Howlader N NA, Krapcho M, Miller D, Brest A, Yu M, Ruhl J, Tatalovich Z, Mariotto A, Lewis DR, Chen HS, Feuer EJ, Cronin KA (eds). SEER Cancer Statistics Review, 1975–2016, National Cancer Institute. Bethesda, MD, https://seer.cancer.gov/csr/1975_2016/, based on November 2018 SEER data submission, posted to the SEER web site, April 2019.

[21] Shallis RM, Wang R, Davidoff A, Ma X, Podoltsev NA, Zeidan AM. Epidemiology of the classical myeloproliferative neoplasms: The four corners of an expansive and complex map. Blood Reviews. 2020;42(100706. 32517877 10.1016/j.blre.2020.100706

[22] Sweet KL, Cortes JE, Apperley JF, Project Confirm: Accelerated Drug Approvals for Chronic Myeloid Leukemia. Clin Cancer Res. 2023;29(12):2179–2183. 36547666 10.1158/1078-0432.CCR-22-2628 PMC10272032

[23] Penna D. New Horizons in Myeloproliferative Neoplasms Treatment: A Review of Current and Future Therapeutic Options. Medicina (Kaunas). 2021;57(11):10.3390/medicina57111181 PMC8619471 34833399

[24] Surveillance, Epidemiology, and End Results (SEER) Program, SEER*Stat Database: Incidence - SEER Research Plus Data, 17 Registries, Nov 2023 Sub (2000–2021) - Linked To County Attributes - Time Dependent (1990–2022) Income/Rurality, 1969–2022 Counties, National Cancer Institute, DCCPS, Surveillance Research Program, released April 2024, based on the November 2023 submission. [cited; Available from: www.seer.cancer.gov

[25] Anderson LA, Duncombe AS, Hughes M, Mills ME, Wilson JC, McMullin MF. Environmental, lifestyle, and familial/ethnic factors associated with myeloproliferative neoplasms. Am J Hematol. 2012;87(2):175–182. 22076943 10.1002/ajh.22212

[26] Titmarsh GJ, Duncombe AS, McMullin MF, How common are myeloproliferative neoplasms? A systematic review and meta-analysis. American Journal of Hematology. 2014;89(6):581–587. 24971434 10.1002/ajh.23690

[27] Sasaki K, Haddad FG, Short NJ, Outcome of Philadelphia chromosome-positive chronic myeloid leukemia in the United States since the introduction of imatinib therapy-The Surveillance, Epidemiology, and End Results database, 2000–2019. Cancer. 2023;129(23):3805–3814. 37769040 10.1002/cncr.35038 PMC11915496

[28] Ma X, Vanasse G, Cartmel B, Wang Y, Selinger HA. Prevalence of polycythemia vera and essential thrombocythemia. American Journal of Hematology. 2008;83(5):359–362. 18181200 10.1002/ajh.21129

[29] Ma X, Vanasse KGJ, Selinger HA, Wang Y, Cartmel B. Prevalence of Polycythemia Vera and Essential Thrombocythemia. Blood. 2007;110(11):4667–4667. 10.1002/ajh.21129 18181200

[30] Mesa RA, Mehta J, Wang H, Epidemiology of Myeloproliferative Disorders in US - a Real World Analysis. Blood. 2012;120(21):2834.

[31] Kantarjian H, O’Brien S, Jabbour E, Improved survival in chronic myeloid leukemia since the introduction of imatinib therapy: a single-institution historical experience. Blood. 2012;119(9):1981–1987. 22228624 10.1182/blood-2011-08-358135 PMC3311242

[32] Vardell VA, Sobieski CE, Tantravahi SK. Trends in Frontline Treatment and Overall Survival in the Era of Targeted Tyrosine Kinase Inhibitor Therapy for Chronic Myeloid Leukemia. Blood. 2022;140(Supplement 1):6763–6764.

[33] Hultcrantz M, Kristinsson SY, Andersson TM, Patterns of survival among patients with myeloproliferative neoplasms diagnosed in Sweden from 1973 to 2008: a population-based study. J Clin Oncol. 2012;30(24):2995–3001. 22802311 10.1200/JCO.2012.42.1925 PMC3417050

[34] Machherndl-Spandl S, Hannouf S, Nikoloudis A, Improved Outcomes in Myelofibrosis after Allogeneic Stem-Cell Transplantation in the Era of Ruxolitinib Pretreatment and Intensified Conditioning Regimen-Single-Center Analysis. Cancers (Basel). 2024;16(19):10.3390/cancers16193257 PMC11482566 39409879

[35] Masarova L, Bose P, Pemmaraju N, Improved survival of patients with myelofibrosis in the last decade: Single-center experience. Cancer. 2022;128(8):1658–1665. 35077575 10.1002/cncr.34103 PMC11963254

[36] Tefferi A, Betti S, Barraco D, Gender and survival in essential thrombocythemia: A two-center study of 1,494 patients. Am J Hematol. 2017;92(11):1193–1197. 28795425 10.1002/ajh.24882

[37] Geyer HL, Kosiorek H, Dueck AC, Associations between gender, disease features and symptom burden in patients with myeloproliferative neoplasms: an analysis by the MPN QOL International Working Group. Haematologica. 2017;102(1):85–93. 27540137 10.3324/haematol.2016.149559 PMC5210236

[38] Tefferi A, Guglielmelli P, Lasho TL, Mutation-enhanced international prognostic systems for essential thrombocythaemia and polycythaemia vera. Br J Haematol. 2020;189(2):291–302. 31945802 10.1111/bjh.16380

[39] Tefferi A, Loscocco GG, Rokach L, Triple A Plus (AAA (+)) Survival Prediction Model for Essential Thrombocythemia: Analysis Involving 7308 Patients. Am J Hematol. 2025;100(11):2017–2027. 40899793 10.1002/ajh.70065 PMC12516648

[40] Passamonti F, Thiele J, Girodon F, A prognostic model to predict survival in 867 World Health Organization-defined essential thrombocythemia at diagnosis: a study by the International Working Group on Myelofibrosis Research and Treatment. Blood. 2012;120(6):1197–1201. 22740446 10.1182/blood-2012-01-403279

[41] Tefferi A, Loscocco GG, Farrukh F, A globally applicable “triple A” risk model for essential thrombocythemia based on Age, Absolute neutrophil count, and Absolute lymphocyte count. Am J Hematol. 2023;98(12):1829–1837. 37665758 10.1002/ajh.27079

[42] Penna D, Lasho TL, Finke CM, 20+ Years and alive with primary myelofibrosis: Phenotypic signature of very long-lived patients. Am J Hematol. 2019;94(3):286–290. 30516867 10.1002/ajh.25351

[43] Stein BL, Williams DM, Wang NY, Sex differences in the JAK2 V617F allele burden in chronic myeloproliferative disorders. Haematologica. 2010;95(7):1090–1097. 20133898 10.3324/haematol.2009.014407 PMC2895032

[44] Spivak JL, Considine M, Williams DM, Two clinical phenotypes in polycythemia vera. N Engl J Med. 2014;371(9):808–817. 25162887 10.1056/NEJMoa1403141 PMC4211877

[45] Karantanos T, Chaturvedi S, Braunstein EM, Sex determines the presentation and outcomes in MPN and is related to sex-specific differences in the mutational burden. Blood Adv. 2020;4(12):2567–2576. 32542392 10.1182/bloodadvances.2019001407 PMC7322953

[46] Karantanos T, Gondek LP, Varadhan R, Gender-related differences in the outcomes and genomic landscape of patients with myelodysplastic syndrome/myeloproliferative neoplasm overlap syndromes. Br J Haematol. 2021;193(6):1142–1150. 34028801 10.1111/bjh.17534 PMC8217263

[47] Khan I, Shergill A, Saraf SL, Outcome Disparities in Caucasian and Non-Caucasian Patients With Myeloproliferative Neoplasms. Clinical Lymphoma Myeloma and Leukemia. 2016;16(6):350–357. 10.1016/j.clml.2016.02.036 27052852

[48] Peseski AM, Saliba AN, Althouse SK, Sayar H. Does race play a role in complications and outcomes of Philadelphia chromosome-negative myeloproliferative neoplasms? Hematology/Oncology and Stem Cell Therapy. 2021; 10.1016/j.hemonc.2021.01.005 33607101

[49] Srour SA, Devesa SS, Morton LM, Incidence and patient survival of myeloproliferative neoplasms and myelodysplastic/myeloproliferative neoplasms in the United States, 2001–12. Br J Haematol. 2016;174(3):382–396. 27061824 10.1111/bjh.14061 PMC4961550

[50] Yan J, Hammami MB, Wei JX, Socio-demographic determinants of myelofibrosis outcomes in an underserved center and the SEER national database. Ann Hematol. 2024;103(9):3543–3551. 39046510 10.1007/s00277-024-05894-7 PMC11358356

[51] Palmer A, Rauscher G, Schoen A, Assessing Disparities in a Chicagoland Cohort of Patients with Myelofibrosis. Blood. 2024;144(3794.

[52] Duncombe AS, Anderson LA, James G, Modifiable Lifestyle and Medical Risk Factors Associated With Myeloproliferative Neoplasms. Hemasphere. 2020;4(1):e327. 32072143 10.1097/HS9.0000000000000327 PMC7000482

[53] Parasuraman SV, Naim AB, Paranagama DC, Financial Burden of Myeloproliferative Neoplasms on Patients: Results from the MPN Landmark Survey in the United States. Blood. 2015;126(23):5561.

[54] Mesa R, Miller CB, Thyne M, Impact of Myeloproliferative Neoplasms (MPNs) on Patients’ Overall Health and Productivity: Results from the MPN LANDMARK SURVEY in the United States. Blood. 2014;124(21):3183.25202141

[55] Goel S, Paoli C, Iurlo A, Socioeconomic burden of participation in clinical trials in patients with myeloproliferative neoplasms. European Journal of Haematology. 2017;99(1):36–41. 28370510 10.1111/ejh.12887

[56] Sasaki K, Haddad FG, Short NJ, Outcome of Philadelphia chromosome-positive chronic myeloid leukemia in the United States since the introduction of imatinib therapy—The Surveillance, Epidemiology, and End Results database, 2000–2019. Cancer. 2023;129(23):3805–3814. 37769040 10.1002/cncr.35038 PMC11915496

[57] Khoury JD, Solary E, Abla O, The 5th edition of the World Health Organization Classification of Haematolymphoid Tumours: Myeloid and Histiocytic/Dendritic Neoplasms. Leukemia. 2022;36(7):1703–1719. 35732831 10.1038/s41375-022-01613-1 PMC9252913

[58] Alvarez-LarrÆn A, Ancochea A, Garc a M, WHO-histological criteria for myeloproliferative neoplasms: reproducibility, diagnostic accuracy and correlation with gene mutations and clinical outcomes. Br J Haematol. 2014;166(6):911–919. 24957246 10.1111/bjh.12990

[59] Sant M, Allemani C, Tereanu C, Incidence of hematologic malignancies in Europe by morphologic subtype: results of the HAEMACARE project. Blood. 2010;116(19):3724–3734. 20664057 10.1182/blood-2010-05-282632

[60] Mehta J, Wang H, Iqbal SU, Mesa R. Epidemiology of myeloproliferative neoplasms in the United States. Leuk Lymphoma. 2014;55(3):595–600. 23768070 10.3109/10428194.2013.813500

